# Principles and Protocols For Post-Cryopreservation Quality Evaluation of Stem Cells in Novel Biomedicine

**DOI:** 10.3389/fphar.2022.907943

**Published:** 2022-05-03

**Authors:** Jingxian Xie, Marlene Davis Ekpo, Jian Xiao, Hongbin Zhao, Xiaoyong Bai, Yijie Liang, Guang Zhao, Dong Liu, Songwen Tan

**Affiliations:** ^1^ Department of Pharmacy, Xiangya Hospital, Central South University, Changsha, China; ^2^ Xiangya School of Pharmaceutical Sciences, Central South University, Changsha, China; ^3^ National Clinical Research Center for Geriatric Disorders, Xiangya Hospital, Central South University, Changsha, China; ^4^ Hunan Carnation Biotechnology Co. LTD, Changsha, China; ^5^ Hainan Nova Doctor Group Co. Ltd, Haikou, China; ^6^ Hunan Sheng Bao Biological Technology Co., Ltd (in Yinfeng Biological Group., Ltd), Changsha, China

**Keywords:** stem cells, quality control, safety evaluation, cryopreservation, cell-based therapy

## Abstract

Stem cell therapy is a thriving topic of interest among researchers and clinicians due to evidence of its effectiveness and promising therapeutic advantage in numerous disease conditions as presented by novel biomedical research. However, extensive clinical application of stem cells is limited by its storage and transportation. The emergence of cryopreservation technology has made it possible for living organs, tissues, cells and even living organisms to survive for a long time at deep low temperatures. During the cryopreservation process, stem cell preparations are subject to three major damages: osmotic damage, mechanical damage, and peroxidative damage. Therefore, Assessing the effectiveness and safety of stem cells following cryopreservation is fundamental to the quality control of stem cell preparations. This article presents the important biosafety and quality control parameters to be assessed during the manufacturing of clinical grade stem cell products, highlights the significance of preventing cryodamage. and provides a reference for protocols in the quality control of stem cell preparations.

## 1 Introduction

Stem cells are widely studied in the field of biology, and their potential in regenerative medicine and therapy is receiving increasing attention (Krishnan et al., 2021). According to the National Institutes of Health Research Administration’s Clinical Research Registry (https://www.clinicaltrials.gov/), as of March 2021, a total of 1,216 mesenchymal stem cells (MSC) clinical research projects were registered worldwide, with 251 in China, accounting for 20.7% of the global total. Stem cells have been broadly classified as adult stem cells (ASCs) and Embryonic stem cells (ESCs) depending on their origin (Pilbauerová and Suchánek, 2018). Stem cell preparations are various types of products obtained by isolating and culturing stem cells from human tissues or healthy donor tissues. Stem cells can be obtained from a variety of tissues such as bone marrow (Tajima et al., 2015), adipose (Yang et al., 2010), umbilical cord (Katheria et al., 2020), umbilical blood (Gil-Kulik et al., 2020), skeletal muscle (Schüler et al., 2021), dental pulp, placenta, amniotic fluid and amniotic membrane (Zare et al., 2021). Stem cell therapy has evolved from basic laboratory research to progressive application in difficult clinical conditions (Chu et al., 2020). Stem cell therapy is the process of implanting human stem cells of autologous or allogeneic origin into the human body after *in vitro* manipulation for disease treatment. Such *in vitro* manipulation includes processes such as isolation, purification, expansion, modification of stem cells, establishment of stem cells (lines), induction of differentiation, freezing (cryopreservation) and recovery after freezing (Hao et al., 2020a). The cell preparation techniques and treatment protocols are diverse, complex and specific to each cell type. However, being a novel biotherapeutic product, all stem cell preparations are subject to similar developmental processes ranging from preparation, *in vitro* testing, *in vivo* animal testing, to clinical trials and clinical application via implantation into humans. At each stage of development, relevant studies and quality control used must be performed to ascertain cell quality, safety and biological effects.

Cryopreservation which is basically the storage of biological materials at very low temperatures so as to conserve their viability has become an integral technique in most experimental and clinical protocols including stem cell transplantation (Hao et al., 2020a). Reports obtained from assessing the benefits of cryopreservation in stem cell therapy reveals that the process preserves the quality of the cells from the point of collection, transportation to final implantation without the loss of vital anatomical and physiological properties. In the same vein, it allows ample time for the screening of donors and recipients for markers like human leukocyte antigen (HLA) which may interfere with optimal therapeutic outcome.

**FIGURE 1:**
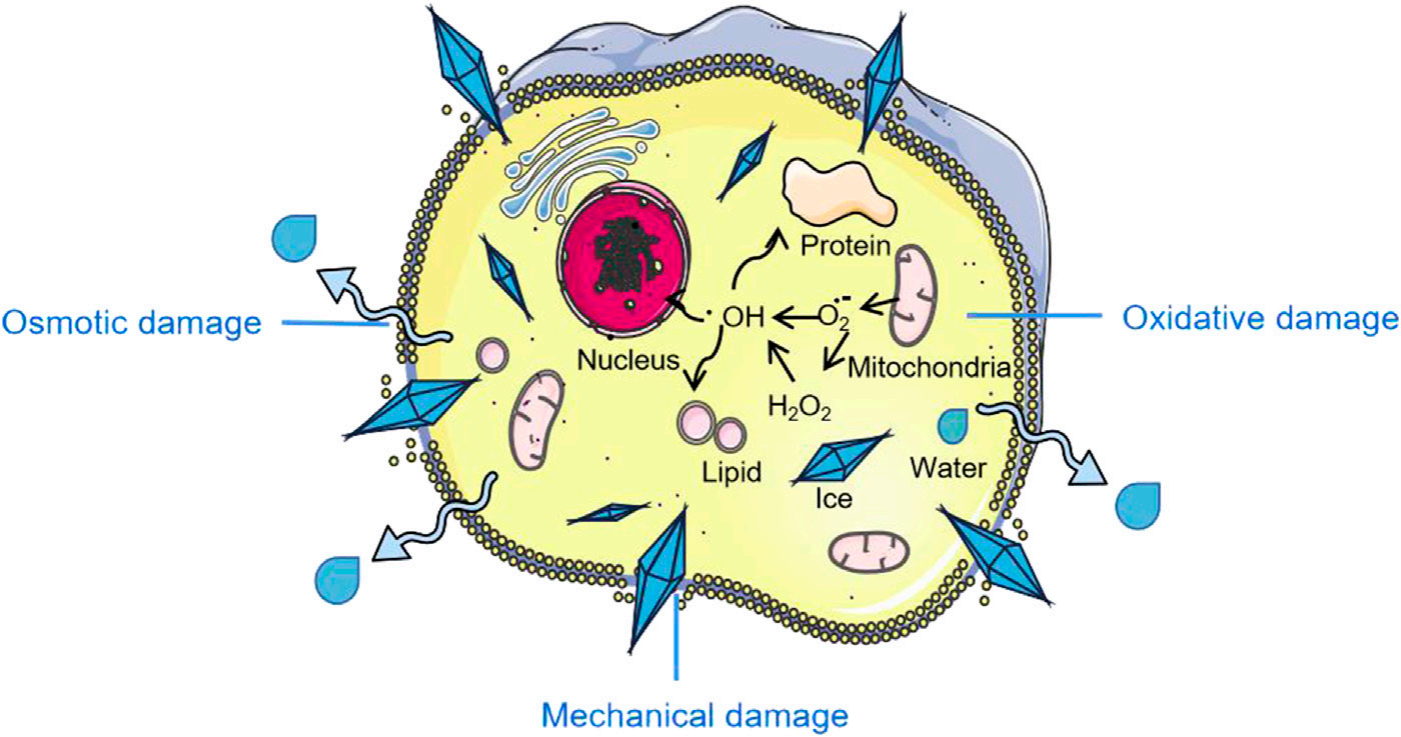
Cryopreservation induced stem cell damage. Parts of the figure are adapted from SMART–Servier Medical Art, Servier: https://smart.servier.com.

Also, multiple passages during culturing and aging of stem cells can lead to reduced cell differentiation potential and genetic alterations (Martin-Piedra et al., 2014), of which cryopreservation becomes the reliable technique to apply in preventing these deleterious effects. None the less, cells are susceptible to damage during freezing, and there are three main types of freezing damage: mechanical damage, osmotic damage, and oxidative damage (Pegg, 2015). Mechanical damage is the irreversible damage to cell membranes and organelles caused by the formation of ice crystals from extracellular and intracellular solutes in cells at low temperatures (Yang et al., 2017). When the extracellular fluid freezes, there is an increase in solute concentration, resulting in cell damage by osmotic dehydration. This event is termed osmotic damage (Finger and Bischof, 2018). On the other hand, oxidative damages are caused by reactive oxygen species (ROS) generated during cryopreservation (Evangelista-Vargas and Santiani, 2017). Ideally, avoiding these undesirable effects is the aim of majority of research studies on cryopreservation as these damages often lead to irreversible harm ranging from the loss of vital functions to even cell death (Calmels et al., 2003) (Figure 1). It is therefore extremely important to explore the cryopreservation of stem cells and its preparations for the establishment of medical cell or tissue banks and the development of clinical regenerative medicine. It is also urgent to develop new approaches for stem cell cryopreservation by analyzing the mechanism underlying cell cryoinjury and optimizing the existing deep cryogenic techniques and methods.

Notwithstanding the enormous efforts and advancements in cancer research, Cancer is persistently among the high mortality diseases where the therapeutic efficacy of conventional chemotherapeutics is limited by factors including toxic side effects and the reoccurrence of tumor. To this end, researchers are on deck seeking to discover better approaches in cancer treatment. One of such is stem-cell based therapy which capitalizes on the desirable features of various stem cells with possible modifications to enhance their anti-tumor potential (Han et al., 2018). In addition to being the active therapeutic agents, stem cells can serve as drug carriers in targeted delivery (Wang et al., 2018), be applied in immuno-modulation following radio (chemo) therapy (Abraham et al., 2022), replace damaged organs through tissue regeneration (Grayson et al., 2015), and provide suitable models for research to aid better understanding and development of novel cancer therapies (Jo H et al., 2021).

This paper summarizes the major cryodamages encountered during the cryopreservation of stem cells and approaches used to tackle them, discusses the factors responsible for cytotoxicity of stem cell preparations in clinical applications, their biosafety concerns and recent techniques used in quality evaluation. This article also provides reference for the quality testing protocols used in the quality control of stem cell preparations, and suggests directions to consider for future quality control and cryopreservation research.

## 2 Cryopreservation of Stem Cells

Cryoinjury is the irreversible damage that cells may suffer during freezing or thawing process (Ross-Rodriguez et al., 2010), and is mainly classified as osmotic damage, mechanical damage, and oxidative damage. In osmotic damage, the extracellular ice formed during slow freezing causes an osmotically driven removal of water from the intracellular space. The resulting hypertonicity is capable of causing cell death (Yousefian et al., 2018). Mechanical and structural cell damage occurs when the cells are cooled rapidly giving insufficient time for intracellular fluid to exit the cells. As a result, the cell experiences detrimental ice nucleation and recrystallization (Bakhach, 2009). Lastly, Oxidative damage is the injury inflicted by ROS produced during cryopreservation, often amounting to the oxidation of lipids, proteins and nucleic acids (Chen and Li, 2020; Liu et al., 2021a) (Figure 1).

Conventionally, the steps in stem cell cryopreservation are as follows: the stem cells of interest are harvested, carefully washed and resuspended in suitable media, then cryopreservation solution containing one or more cryoprotectants (CPAs) is then added (Wang et al., 2022). Till date, Dimethyl sulfoxide (DMSO) is the CPA of choice in stem cell cryopreservation commonly applied at a final concentration of 5–10% (v/v) (Yamatoya et al., 2022). The vial containing the cells and survival promoting additives is later frozen to −80°C using controlled rate freezers usually operated at optimal cooling rates of −1 to −3°C per minute. Finally, the frozen sample is placed in a liquid nitrogen tank mostly at −196°C for long-term preservation (Calmels et al., 2003). On demand, the stem cells are unfrozen by rapid thawing in a in a 37°C water bath and the CPA is removed before administration to patients. Adverse drug reaction monitoring following stem cell implantation procedures has documented some unwanted reactions including symptoms like abdominal cramps, diarrhea, nausea, flushing, and life-threatening events such as acute renal failure, respiratory depression and cardiac arrhythmias (Shu et al., 2014). Some of the observed adverse effects has been linked to toxicity caused by traces of DMSO retained in the infusion (Ataseven et al., 2017; Maral et al., 2018). Although the mechanisms underlying the adverse effects from infusing cryopreserved stem cell products are not completely unraveled, other contributing factors besides the toxicity of DMSO include cell aggregation and presence of apoptotic debris (Schlegel et al., 2009), volume of infused suspension and presence of unnucleated cells (Rohner et al., 2019), hypothermic condition of the cell suspension, and electrolyte imbalance (Calmels et al., 2003).

Predictably, decreasing the quantity of DMSO added during cryopreservation would diminish unwanted side effects. CPAs can be classified as permeable and non-permeable based on their ability or inability to cross the cellular membrane. Generally, permeable CPAs like DMSO are more toxic compared to non-permeable CPAs at equivalent concentrations (Raju et al., 2021). Several studies have shown that the inclusion of trehalose (Zhang et al., 2003), sucrose (Pan et al., 2017), polyampholytes (Matsumura et al., 2010) and antifreeze proteins (AFPs) (Shaliutina-Kolešová et al., 2019) during cryopreservation can greatly reduce the working concentration of DMSO, thereby reducing the cytotoxicity caused by DMSO. Another potent strategy is to develop alternatives to DMSO which should ideally prevent cryodamage and promote survival of the cryopreserved material and concomitantly be biocompatible. CPAs like trehalose and sucrose are suitable candidates as they were found to promote cryopreservation outcome in hematopoietic stem cells by maintaining the CD45+/34+ cell population and retaining cell clonogenicity and viability (Rodrigues et al., 2008).

## 3 Key Aspects of Quality Control of Stem Cell Preparation

Stem cell products should be generated in compliance with Good Manufacturing Practices (GMP). There are no uniform global guidelines for the production and clinical application of stem cell products. The United States Food and Drug Administration (USFDA), the European Medicines Agency (EMA), the Japanese Pharmaceuticals and Medical Devices Agency (PMDA), and other regulatory agencies currently provide GMPs to promote the safe use of therapies for patients. In addition, China has successively proposed general requirements for stem cells, which is applicable to stem cell research and production (Hao et al., 2020a; Hao et al., 2020b; Zhang et al., 2022). The following are the key aspects of quality control of stem cell preparation summarized in Table 1.

**TABLE 1:** Routine quality evaluation techniques in stem cell quality control.

Quality Assessed	Aim	Parameter Assessed	Method	Requirements	REF
Identity/Purity	Comprehensive cellular identification of different donors and different types of stem cells	Cell morphology	Electron microscope	Cells grown in 2D conditions shall exhibit growth as colonies with clear boundaries, high nuclear-cytoplasmic ratios and uniform morphology. Within each colony, cell-cell contact should be tight. Zhang et al. (2022)	Gherghiceanu and Popescu (2010)
		Genetics	RT-qPCR, Flow cytometry	ND	Jo HY et al. (2021)
			Single-Cell RNA Sequencing		
		Surface markers	Flow cytometry	Cell surface markers: ≥70.0% of the cell population express any two of the following genes: SSEA3, SSEA4, TRA-1-60, TRA-1-81, for example, TRA1-81–positive rate ≥70.0% and SSEA4–positive rate ≥70.0%; intracellular markers: OCT4-positive rate ≥70.0% and NANOG-positive rate ≥70.0%	Jo HY et al., (2021), Zhang et al. (2022)
		Specific gene expression products	WB	ND	Zhang et al. (2022)
			RT-qPCR		
Viability	Testing for cell activity and growth status	Survival rate	Trypan blue staining	Cell viability shall be ≥ 90% before cryopreservation, and ≥60% post-thaw.	Kamalifar et al. (2020), Zhang et al. (2022)
			MTT		
		Telomerase activity	PCR, High-resolution optical tweezers	ND	Patrick et al. (2020)
		Cell proliferation rate	MTT	ND	Kamalifar et al. (2020)
		Cell cycle	Flow cytometer, Fluorescence Detection	ND	Fendrik et al. (2019)
		Clone forming efficiency	Trypan blue staining	ND	Dutta et al. (2011)
			Flow cytometer		
		Organelle activity	Catalase activity	ND	Liu et al. (2021b)
			Membrane potential		
			Naþ/Kþ-ATPase, Ca2þ/Mg2þ-ATPase		
		Integrity of mitochondrial DNA	ND	ND	Yahata et al. (2017)
		Exosomes	TEM and immunoblotting	ND	Ain et al. (2018)
Sterility	Testing for the presence of mycoplasma, bacterium and fungi	Mycoplasma; bacterium; fungi	Turbidity testing, Chemosensitivity testing, MALDI-TOF MS	Negative	Golay et al. (2018), Hao et al. (2020b), Hartnett et al. (2021)
Adventitious viruses	The viruses to detect should be evaluated case by case by risk analysis	HBV、HCV、HCMV、HIV、HSV、TP, etc	PCR, Cytopathic effect	Negative	Hao et al. (2020b), Fernandez-Muñoz et al. (2021)
Endotoxins	Elimination of the effects of endotoxins	Endotoxins	Limulus amebocyte lysate	<2 EU/ml	Guo et al. (2011)
					Nomura et al. (2018)
Tumorigenicity	Avoiding Tumors from Stem Cell Therapy	Animal experiments	*In vivo* tumor formation assay in athymic mice	ND	Gowing et al. (2014)
		Tumorigenic transformed cells	ND	ND	Sato et al. (2019)
		Karyotype	Resuscitating and culturing samples for 48–72 h prior to cell harvesting and karyotyping.	46, XY, or 46, XX.	Andrews et al. (2017); Hao et al. (2020b),
		CGH array	CGH	ND	Andrews et al. (2017)
		Colony-forming assays in soft agar	ND	ND	Fernandez-Muñoz et al. (2021)
		migration rate	Scratch test	ND	Matluobi et al. (2018)
Potency	Determining the biological effectiveness of stem cell preparations in relation to therapy	Tri-lineage differentiation potential	ND	ND	Fernandez-Muñoz et al. (2021)
		Cytokines	MB-FIA	ND	Sane et al. (2018)
		Differentiation potential	ELISA, RT-PCR	ND	Matluobi et al. (2018)
		Specific genes and proteins	WB, ELISA	ND	Kang et al. (2019)
DNA Fingerprint	To distinguish the origin of stem cells after transplantation	DNA Fingerprint	STR, VNTR	ND	Blau et al. (1999)
Stem cell-related genes	To assist in assessing the activity and effectiveness of stem cells	Anti-oncogene	PCR	ND	Huang et al. (2013)
		Proto-oncogene	PCR	ND	Daekee et al. (2019)
		Stem cell-related genes (Oct3/4, Nanog, Sox2)	PCR, NGS	ND	Mendes Oliveira et al. (2018)
					Daekee et al. (2019)
Culture medium	Removal of residual ingredients	Bovine serum protein, antibiotics, cytokines	ELISA	ND	Zhang et al. (2011)

ND: Not determined, VNTR: variable number of tandem repeats, ELISA: enzyme linked immunosorbent assay, PCR: Polymerase chain reaction, WB: Western blot, CGH: Comparative Genomic Hybridization, RT-PCR: reverse transcription PCR, MB-FIA: Magnetic-bead fluorescent immunoassays, MS: mass spectroscopy, MALDI-TOF: Matrix-assisted laser desorption/ionization-time of flight MS, NGS: next generation sequencing, TEM: transmission electron microscopy, HBV: Hepatitis B Virus, HCV: Hepatitis C Virus, HCMV: Human Cytomegalo virus, HIV: Human immunodeficiency virus, HSV: Herpes simplex virus, TP: treponema pallidum.

### 3.1 Analysis of Cell Characteristics

Some important methods used to characterize and identify stem cell types include short tandem repeat (STR) typing, morphological examinations, identification of markers, analysis of *in vitro* differentiation and teratoma formation (Zhang et al., 2022). As made known by science, there is yet a great number of undiscovered truths in stem cell research. For instance, new stem cell markers are being identified which could be beneficial in understanding and classifying stem cells. Also, the variations observed in expression of stem cell markers has been attributed to cell isolation and culturing techniques, cell age, culture medium composition, and stage of cell differentiation. Tapia et al. reports varied cell differentiation and expression of stemness markers in human induced pluripotent stem cells (hiPSCs) cells generated with different reprogramming methods (Tapia and Schöler, 2016). Also, the presence of certain growth factors and removal of oxygen from the growth media has induced alterations in stem cell markers (Hagmann et al., 2013).

### 3.2 Analysis of Biological Safety

#### 3.2.1 Tumorigenicity

The tumorigenicity of stem cells is one of the main factors hindering their clinical use (Yasuda and Sato, 2015). Tumors can be generated through a number of pathways one of which is the malignancy of stem cells induced during proliferation and differentiation (Miyawaki et al., 2017). Additionally, teratomas can originate from undifferentiated stem cells, especially for human induced pluripotent stem cells (hiPSCs) (Yuan, 2015).

#### 3.2.2 Sterility

The microbial contamination of stem cell products (Hartnett et al., 2021) including bacterial, fungal, mycoplasma and bacterial endotoxin contaminations is responsible for a significant number of adverse reaction cases (Golay et al., 2018). Therefore, microbiological testing prior to clinical application is pertinent for preventing unwanted effects. The recently developed and frequently applied BacT/Alert 3D automated culture system is one of the innovations to aid fast and efficient sterility testing; it requires short incubation period and does not differ significantly from pharmacopeial methods in terms of detection capacity (England et al., 2019). Rapid mycoplasma detection methods include nucleic acid amplification technology and mycoplasma metabolic enzyme activity detection (Zhang et al., 2022).

#### 3.2.3 Pathogenic Factors

A combination of *in vivo* and *in vitro* methods should be used to test for human- and animal-derived specific pathogenic factors based on the characteristics of each stem cell preparation. If bovine serum has been used, testing for bovine-derived specific viruses shall be performed; if pig-derived materials such as trypsin are used, testing for at least pig-derived microviruses shall be performed (Zhang et al., 2022).

### 3.3 Analysis of Biological Activity

The biological effectiveness of various types of stem cells can be basically categorized based on their ability to induce differentiation, immunomodulatory ability and tissue regeneration (Li et al., 2021). Biological potency assay include: secretion of relevant bioactive substances (Matluobi et al., 2018) (e.g., recombinant proteins, glycoproteins or lipoproteins, growth factors, enzymes and cytokines), extracellular matrix/structures, cellular interactions (e.g., immune activation or inhibition), migration differentiation or self-renewal potential of cells (Hao et al., 2020a). To evaluate the immune activity of stem cells, (a combination of) methods like quantitative ribonucleic acid (RNA) analysis, marker assays, secreted protein analysis and immune cell response analysis have been suggested. Furthermore, instrumentations such as immunofluorescence staining, morphological observation, flow cytometry analysis and electrophysiological analysis are applicable in this respect (Rohner et al., 2019).

For MSCs, regardless of their origin, the differentiation ability of multiple cell types (e.g., adipocytes, chondrocytes, osteoblasts, etc.) should be tested *in vitro* to determine their multipotency of cell differentiation (Lee et al., 2015). For undifferentiated ESCs and iPSCs, the pluripotency of cell differentiation must be measured by their ability to form embryoid bodies *in vitro* or teratomas in severe combined immunodeficiency disease (SCID) mice model (Li et al., 2013). In addition to this, biological effects tests relevant to confirming the intended therapeutic activity of stem cells should be performed as specific biological activity assays.

## 4 Future Research and Directions For Quality Control

### 4.1 Efficient Cryopreservation Solutions

The use of efficient and low toxicity cryopreservation protocols to reduce cryogenic damage would guarantee the effectiveness of stem cell preparation applications post-thaw. Since different cryoprotectants have different protection mechanisms, a mixture of cryoprotectants is usually used to maximize cell survival (Tao et al., 2020). The combination of cryoprotectants can reduce the concentration of a single CPA used, thus reducing cytotoxicity (Elliott et al., 2017). The addition of trehalose (Yamatoya et al., 2022), sucrose (Shu et al., 2014), polyampholytes (Maral et al., 2018) and antifreeze proteins (Shaliutina-Kolešová et al., 2019) during cryopreservation can greatly minimize the required amount of DMSO, thereby reducing cytotoxicity.

### 4.2 Elimination of Residual Undifferentiated Stem Cells

Residual undifferentiated hiPSCs pose tumorigenic risk, and methods to eliminate these undifferentiated hiPSCs are crucial to ensuring safety (Blum and Benvenisty, 2008). The introduction of suicide genes (Yagyu et al., 2015), addition of plasma-activated medium (Matsumoto et al., 2016), cell sorting using antibodies against hiPSC surface antigens11 and, the use chemical inhibitors (Ben-David et al., 2013) are useful techniques for separating undifferentiated stem cells. Nevertheless, none of these methods have attained application in clinical grade stem cell production due to high cost, low specificity and retainment of residue in the final product (Mao et al., 2017). Consequently, researchers are beset with the task of discovering new effective ways to eliminate undifferentiated hiPSCs. In this light, Takunori et al. presented in 2018 evidence supporting the ability of high L-alanine concentrations to selectively eliminate undifferentiated hiPSCs through a novel pathway (Nagashima et al., 2018).

This method may contribute to the development of a low-cost, safe, and practical method to eliminate residual undifferentiated hiPSCs.

### 4.3 The Integrity of Mitochondrial DNA in Pluripotent Stem Cells

Mitochondrial DNA mutations occur at a high rate, causing several debilitating and life-threatening diseases like Kearns–Sayre syndrome and Pearson syndrome (Greaves and Taylor, 2006). According to Prigione et al., pluripotency caused mitochondrial DNA mutations originally absent from the parent PSCs (Prigione et al., 2011). Furthermore, fluctuations in the number of mitochondrial copies have been detected during prolonged culturing of iPSCs isolated from donors presenting with different maladies like lactic acidosis, mitochondrial myopathy, encephalopathy and stroke-like episodes (Gherghiceanu and Popescu, 2010). These reports confirm the need to conduct more research geared at identifying other implacable factors, understanding the mechanism (s) behind these DNA alterations and also to assess the integrity of mitochondrial DNA and genomic DNA before the use of stem cells in humans.

### 4.4 New Cytogenetic Techniques

Presently, an array of sensitive cytogenetic tools such as fluorescent *in situ* hybridization, comparative genomic hybridization, Giemsa (GTG) karyotyping and whole genome sequencing have been developed to assess the genomic integrity of stem cells (Rohani et al., 2018). Single-cell genome sequencing can rapidly and efficiently detect genetic heterogeneity in large cell samples (Bardy et al., 2016). Single-cell RNA-seq has been used in combination with electrophysiology to evaluate the activity of human iPSCs derived neurons (Bardy et al., 2016). Similarly, single cell sequencing has been applied in the long-term monitoring of trends in cancer development, progression and diversity among populations (Navin, 2015). A collaborative paper published by the International Stem Cell Banking Initiative point out the importance of stem cell genetic integrity, and also mention that other cytogenetic techniques like FISH, SKY, CGH arrays, and whole-genome sequencing would be useful to identify information that GTG karyotyping cannot acquire (Andrews et al., 2015). It is unclear how essential it is to perform more thorough epigenetic screening of pluripotent stem cells prior to clinical application. Report of variable loss of genomic imprinting across lines in iPSCs suggest that standardized epigenetic quality control tests would be beneficial (Pasque et al., 2018).

## 5 Conclusion

Quality control of stem cells and stem cell-based medicines have received widespread attention, and the development of cryopreservation technology has provided a technical guarantee for the application of stem cell preparations. The main risks faced by patients with stem cell preparations are the occurrence of allergic or immune reactions, the formation of tumors, and the occurrence of microbial contamination. These risk-posing factors should be eliminated or reduced to tolerable limits in the preclinical stages by extensive *in vivo/in vitro* evaluation to improve the efficacy and safety of stem cells. To ensure the safety and efficacy of stem cell products, each batch of stem cell preparation shall meet the existing stem cell quality requirements covering cell identification, viability and growth activity, purity and homogeneity, sterility testing and mycoplasma testing, detection of endogenous pathogenic factors, endotoxin testing, abnormal immunological response, tumorigenicity, biological potency testing, residual amount of culture medium and other added components. These strict standards of quality control must be adhered to before stem cells can be used for clinical applications, and such standards must be harmonized and monitored globally to ensure uniformity in the grade of clinically applicable stem cell products. Advanced techniques like single-cell genome sequencing of large samples may provide better understanding of genomic integrity in stem cell lines. Also, these evaluations should be conducted post-cryopreservation during the formulation and testing phases to ensure there is no cryodamage. Furthermore, cryopreservation protocols should be reviewed and tailored to each stem cell, putting into key consideration its efficacy and safety. In any case, more clinical resources and research studies should be targeted at further optimizing the quality control of stem cells before venturing further into application of stem cell therapies.

## Data Availability

All data used to support the findings of this review are included within the article.
