# Supplementary material for: Principles and Protocols For Post-Cryopreservation Quality Evaluation of Stem Cells in Novel Biomedicine
Source: Front Pharmacol. 2022 May 3;13:907943. doi: 10.3389/fphar.2022.907943 (PMC9113563; doi:10.3389/fphar.2022.907943)
Supplement: Supplementary file 1 [file DataSheet1.docx]

***Supplementary Information***

**Cell viability test(1, 2)(1) (2)**

1.Preparation of cell suspension

Harvest and suspend the cells with appropriate volume of DPBS. The cells in the haemocytometer shall be 20-50 cells/mm2. Serial dilution is necessary if the number of cells exceeds 200 per haemocytometer.

2. Trypan blue staining

Evenly mix the Trypan blue solution with the cell suspension at a volume ratio of 1:1.

3.Cell counting

Load the haemocytometer with 10 μL of the trypan blue-labelled sample. Make sure the entire chamber is filled with the testing sample. Stand for 30 seconds, count the stained cells and the total number of cells respectively.

1. Calculation and analysis

S = (M− D)∕M × 100%.

In the equation: S——viability of cells M——total number of cells D——number of stained cells

**Detection of cell markers(Flow cytometry)(3) (1)(2, 3)**

1.Sample preparation and fixation

Collect samples by centrifuging single cell suspensions at 250 g for 3 minutes.Discard the supernatant. Resuspend the cells in an appropriate volume of fixing solution and incubate for 10 minutes in an ice bath. Wash the cell samples with an appropriate volume of wash solution for 3-5 times (3-5 minutes each time).

2.Blocking and permeabilization

Resuspend the fixed sample with the blocking/permeabilization solution and aliquot the cells into two independent samples, which will be used as a testing sample and an isotype control sample respectively. Incubate on ice for 20 minutes, then wash the samples with the wash solution.

3.Antibody incubation

4.Filtering and loading

Resuspend the samples with wash solution and then transfer the cell suspension into flow cytometry tube by filtering the samples through a mesh with 40μm pores. Load the samples into the flow cytometer and perform testing according to the manufacturer's instruction.

1. Gating

Gate the target population of cells based on particle size and transparency, excluding cell debris and other irrelevant particles. The gating of positive staining cells shall be determined by the fluorescence intensity using isotype controls as a reference. Both positive and negative experimental controls shall be set up for gating and the following analysis.

**Quantitative PCR analysis(1) (4)**

Reagents:Commercial kit for quantitative PCR;Commercial genomic DNA extraction kit;PCR primers for the target genes

**Normative references**

Most of the normative references listed in the main text became effective by publishing as hard copies in China. Here we provide the source information in the table below should readers intend to explore further.

| GB/T6682-2008 Water for analytical laboratory use – specification and test method | Standard Book #155066.1-32760 |
| --- | --- |
| Diagnosis for hepatitis C | http://www.nhc.gov.cn/wjw/s9491/201803/29997c16d2f24e639ab6c6f55105a9d0.shtml |
| Diagnosis for HIV / AIDS | http://www.nhc.gov.cn/wjw/s9491/201905/6430aa653728439c901a7340796e4723.shtml |
| Diagnostic criteria for viral hepatitis B | http://www.nhc.gov.cn/wjw/s9491/200907/41983.shtml |
| General requirements for stem cells | Standard Book #155066.2-32089 |
| Human embryonic stem cell | Standard Book #155066.2-34099 |
| Pharmacopoeia of the People’s Republic of China | ISBN：978-7-5214-1575-9 |
| National Guide to Clinical Laboratory Procedures | ISBN：978-7-1171-9862-2 |

References:

1. Hao J, Cao J, Wang L, Ma A, Chen S, Ding J, et al. Requirements for human embryonic stem cells. Cell Proliferation. 2020;53(12):e12925.

2. Zhang Y, Wei J, Cao JN, Zhang KH, Peng YJ, Deng HK, et al. Requirements for human-induced pluripotent stem cells. Cell Proliferation.

3. Gherghiceanu M, Popescu LM. Cardiomyocyte precursors and telocytes in epicardial stem cell niche: electron microscope images. Journal of Cellular and Molecular Medicine. 2010;14(4).

4. Hao J, Ma AJ, Wang L, Cao JN, Chen S, Wang L, et al. General requirements for stem cells. Cell Proliferation. 2020;53(12).
